# Long COVID Is Not a Functional Neurologic Disorder

**DOI:** 10.3390/jpm14080799

**Published:** 2024-07-29

**Authors:** Todd E. Davenport, Svetlana Blitshteyn, Nicola Clague-Baker, David Davies-Payne, Glenn J. Treisman, Sarah F. Tyson

**Affiliations:** 1 Department of Physical Therapy, University of the Pacific, Stockton, CA 95211, USA; 2 Workwell Foundation, Santa Rosa, CA 95403, USA; 3 Department of Neurology, Jacobs School of Medicine and Biomedical Sciences, University of Buffalo, Buffalo, NY 14203, USA; 4 Dysautonomia Clinic, Williamsville, NY 14221, USA; 5 School of Allied Health Professions and Nursing, Institute of Population Health, University of Liverpool, Liverpool L69 7ZX, UK; 6 Department of Radiology, Starship Children’s Hospital, Auckland 1023, New Zealand; 7 Department of Psychiatry, Johns Hopkins School of Medicine, Baltimore, MD 21287, USA; glenn@jhmi.edu; 8 School of Health Sciences, University of Manchester, Manchester M14 4PX, UK; sarah.tyson@manchester.ac.uk

**Keywords:** post-COVID-19 condition (PCC), post-acute sequalae of COVID-19 (PASC), myalgic encephalomyelitis, chronic fatigue syndrome, neurasthenia, conversion disorder, dysautonomia, neurology, physical examination, imaging

## Abstract

Long COVID is a common sequela of SARS-CoV-2 infection. Data from numerous scientific studies indicate that long COVID involves a complex interaction between pathophysiological processes. Long COVID may involve the development of new diagnosable health conditions and exacerbation of pre-existing health conditions. However, despite this rapidly accumulating body of evidence regarding the pathobiology of long COVID, psychogenic and functional interpretations of the illness presentation continue to be endorsed by some healthcare professionals, creating confusion and inappropriate diagnostic and therapeutic pathways for people living with long COVID. The purpose of this perspective is to present a clinical and scientific rationale for why long COVID should not be considered as a functional neurologic disorder. It will begin by discussing the parallel historical development of pathobiological and psychosomatic/sociogenic diagnostic constructs arising from a common root in neurasthenia, which has resulted in the collective understandings of myalgic encephalomyelitis/chronic fatigue syndrome (ME/CFS) and functional neurologic disorder (FND), respectively. We will also review the case definition criteria for FND and the distinguishing clinical and neuroimaging findings in FND vs. long COVID. We conclude that considering long COVID as FND is inappropriate based on differentiating pathophysiologic mechanisms and distinguishing clinical findings.

## 1. Introduction

Severe fatigue that impairs usual function long has been described throughout recorded human history. The neurologists Beard **[1]** and Charcot **[2]** were among the first to characterize the health condition ‘neurasthenia’ in the latter half of the 19th century. Based on this common historical root in neurasthenia, two divergent scholarly and clinical paths have taken shape over time. The first path involves a pathogenic disease model rooted in the scientific process, resulting in a rich literature describing pathobiology and various attempts at creating specific case definition criteria. This path has resulted in the label of myalgic encephalomyelitis/chronic fatigue syndrome (ME/CFS). The second path is a psychosomatic/sociogenic illness construction that has incorporated ideas from contemporary neuroscience into an unbroken conceptual chain linking back to neurasthenia. This path has resulted in the label of functional neurologic disorders (FND).

Long COVID has caused a renewed scholarly and clinical focus on complex chronic conditions associated with infections [3,4,5,6,7,8,9,10,11,12,13,14,15,16,17,18,19]. According to the National Academy of Science, Engineering, and Medicine definition, long COVID is “an infection-associated chronic condition that occurs after SARS-CoV-2 infection and is present for at least 3 months as a continuous, relapsing and remitting, or progressive disease state that affects one or more organ systems” ([20], p. 2). Long COVID consists of single or multiple symptoms attributable to single or multiple diagnosable conditions [20]. It can follow asymptomatic, mild, or severe SARS-CoV-2 infection [20]. Among individuals with a positive COVID-19 test, approximately 43% of non-hospitalized cases and over half of hospitalized cases report symptoms and signs of long COVID, according to data from the first two years of the pandemic [21]. More recently, testing for SARS-CoV-2 infection has become far less frequent within society and surveillance testing has been discontinued. In addition, many individuals with COVID-19 now convalesce outside the medical system, so these cases are undetected. These observations suggest the prolonged decreases in function and delayed recovery [22,23,24,25] associated with COVID-19 may be undercounted and accelerating over time.

The intensity and disablement of fatigue associated with long COVID is similar to other post-viral conditions, including post-treatment Lyme disease [26], chronic Epstein–Barr infection [27], and post-mononucleosis syndrome [28]. The condition may range from mild impairment of function to severely disabling exhaustion. Patient complaints include severe waxing and waning fatigue, worsening fatigue the day after exertion, and dramatic exacerbation by efforts to exercise. Associated symptoms, including cognitive impairment (often referred to by patients as *brain fog*, diffuse chronic pain, sleep disruption, and autonomic dysfunction, including POTS, migraine, gastrointestinal dysmotility, and temperature intolerance are common concomitants. The onset of symptoms may be continuous from the time of infection or delayed in onset by weeks or months following an apparent full recovery from the acute phase of infection [20]. Long COVID disablement can range from mild to severe, and it can resolve in a period of months, or it can persist and worsen over time. Disablement related to long COVID may result in profound functional impairments in self-care, as well as family, social, school, and occupational roles [20]. Post-exertional malaise/post-exertional neuroimmune exhaustion (PEM/PENE) is common among people with long COVID [25,29,30], which accounts for the persistent, severe, and often progressive pattern of disablement in long COVID. PEM/PENE is a clinical hallmark of ME/CFS, suggesting an ME-like subtype of long COVID is prevalent [25,29,31,32,33]. Therefore, it is perhaps unsurprising that many of the same themes historically characterizing the narrative about ME/CFS are still influencing the discourse surrounding long COVID.

An accumulating body of research indicating the underlying pathophysiology of long COVID involves a complex interaction between processes and systems. Long COVID has been acknowledged to exacerbate pre-existing health conditions, or it may present as new diagnosable health conditions [20]. However, psychosomatic/sociogenic illness constructs continue to influence the contemporary discourse related to long COVID [34]. This clinical perspective will anchor the current discourse regarding long COVID into the historical context involving a parallel development of ME/CFS (predominately pathobiological) and FND (predominately psychosomatic/sociogenic) diagnostic constructs. This perspective will now review the clinical findings and neurobiological pathology of long COVID, developing a clinical and scientific rationale for why it is inappropriate to consider long COVID as FND.

## 2. Pathobiological Disease Characterization: From Neurasthenia to Myalgic Encephalomyelitis/Chronic Fatigue Syndrome

Some neurologists considered neurasthenia as a form of nervous exhaustion that caused severe mental and physical fatigue, even following the mild exertions associated with normal daily functions like self-care, family and community activities, and remunerative work [35]. Weakness characterized by abnormally rapid fatiguability and slow recovery following exertion were classically associated with neurasthenia. Yet, people with neurasthenia generally had unremarkable findings on physical examination despite having often severe functional limitations from a whole constellation of associated signs and symptoms. Nervous system exhaustion, nerve over-excitability, and impaired cerebral blood flow all were implicated as potential patho-etiological factors, perhaps secondary to overwork, toxicity, or infection [35]. The absence of remarkable physical findings consistent with neurasthenia, perhaps combined with a high prevalence of neurasthenia in women, led to early psychological theories suggesting that emotional disturbances corresponding to the severe physical and mental symptoms, signs, and disablement must be causal factors [35].

The personal and societal challenges of persistent fatigue never abated, even as the diagnosis of neurasthenia began to fall out of favor. It was during this time that the association between persistent fatigue and infection began to be more deeply explored. In 1934, Gilliam [36] documented an outbreak of infectious disease that caused lingering signs and symptoms at Los Angeles County General Hospital in the United States. Poliomyelitis was the best-known epidemic at the time, so Gilliam called this new condition atypical poliomyelitis [36]. Outbreaks of atypical poliomyelitis were also documented in Iceland in 1946–1947 and 1948–1949 [37]. In 1955, the term benign myalgic encephalomyelitis (ME) was introduced to describe the post-acute signs and symptoms following infectious disease at the Royal Free Hospital (London, UK) [38,39]. The term *epidemic ME* was then coined at a 1978 symposium of the Royal Society of Medicine [40]. This development was the medical community’s first acknowledgement of ME as a distinct disease process, instead of a behavioral disorder. ME began to reach the popular consciousness in the mid-1980s US following an outbreak of post-infectious illness in Incline Village, Nevada. Work surrounding this outbreak led to assigning the name chronic fatigue syndrome (CFS) to signs and symptoms following an infection [4]. Although clinicians and researchers thought this term best described the phenomenon [41], people with ME believe it poorly represents their lived experience. Unsurprisingly, the term CFS remains deeply unpopular among people living with ME/CFS [42] even as it continues to find a common usage.

The nature of lingering symptoms, signs, and disability was the subject of exploration as the various outbreaks were documented. Ramsay first coined the term epidemic malaise to describe the phenomenon of muscle weakness that was worsened upon repeat testing [38,39]. This observation of a physical performance decline in response to a previous exertion was formative to developing contemporary case definition criteria for ME/CFS. PEM/PENE is now recognized as a whole host of unusual signs and symptoms following exertion, such as profound fatigue, cognitive dysfunction (such as impairment in attention, short-term memory, and performing mental calculations), sleep disturbance, clinical presentations consistent with viral reactivation (such as fevers, swollen glands, and pharyngitis), body and joint pains, headaches, and muscle weakness [43,44,45,46,47,48,49,50]. PEM/PENE appears responsible for the episodic disability observed in people living with ME/CFS. Episodic disability suggests a person’s physical and cognitive abilities may vary substantially within a short term of hours to days (i.e., microcycling) and a long term of weeks, months, and years (i.e., macrocycling) [51,52]. In addition, PEM/PENE has increasingly become a component of case definition criteria over time to differentiate the phenomenon of debilitating fatigue, among other signs and symptoms, after exposure to a pathogen or toxin from other causes of fatigue.

Various case definitions to describe ME have been created throughout the late 20th century and early 21st century. These case definitions include the Holmes et al. [4] criteria (1988), Oxford criteria (1991) [53], Fukuda et al. criteria (1994) [53] and its elaboration by Reeves et al. (2005) [54], Canadian Consensus Criteria (CCC; 2003) [43], International Consensus Criteria for ME (ICC-ME; 2011) [44], criteria for Systemic Exertional Intolerance Disease (SEID; 2015) [55], and the UK National Institute for Health and Care Excellence guideline (UK NICE; 2021) [56]. There has been a progressively increasing prominence for the role of PEM/PENE as an important differentiating factor between ME/CFS and other health conditions associated with fatigue. PEM/PENE is now perhaps the most important specific (rule-in) consideration to identify ME/CFS and distinguish it from other health conditions that involve disabling fatigue.

**Figure 1:**
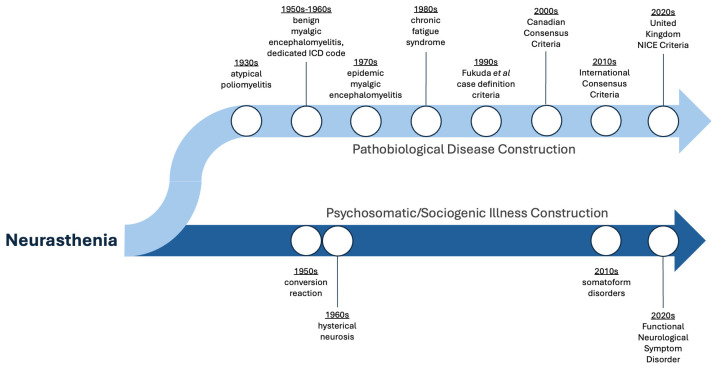
Key time points in the parallel development of disease and illness constructions resulting in myalgic encephalomyelitis/chronic fatigue syndrome (pathobiological illness construction) and functional neurologic disorder (psychosomatic/sociogenic illness construction), based on a common historical root in neurasthenia.

ME was first discussed as being different from other neurological disease processes in a 1956 paper that first used the term “benign myalgic encephalitis” to distinguish it from other infectious encephalitic infections and, perhaps most importantly, hysteria [57]. It was first assigned an International Classification of Diseases (ICD) code in the ICD-8 1969 (code 332) [58]. ME and CFS are included in ICD-11 as post-viral syndromes (8E49) [59]. Inclusion of ME is evidence of improving legitimacy within the biomedical community, as the clinical characteristics and courses of these conditions have become better understood over time. Notably, ME and CFS are not included in the ICD as mental or behavioral disorders [59]. Key points in the development of a pathobiological disease construction resulting in the collective understanding of ME/CFS are summarized in Figure 1.

## 3. Psychosomatic/Sociogenic Illness Construction: From Neurasthenia to Functional Neurologic Disorder

While decades of scientific work have led down the path of iterative case definition criteria and the determining of the underlying pathophysiology of ME/CFS, a parallel path largely has repeated old thinking with new labels (Figure 1). The first edition of the Diagnostic and Statistical Manual (DSM) of the American Psychiatric Association listed hysteria as conversion reaction [60], transitioning to hysterical neurosis in the DSM’s second edition [61]. These titles were based on the early concepts of hysteria as a uterine disorder in women [62]. A major underlying hypothesis advanced by Freud is that hysterical disorders involved the conversion between a somatic symptom and a repressed feeling or idea, such as a somatic symptom arising from anxiety, hence the term conversion disorder [63]. With the transition away from a system classifying disorders based on putative etiology and toward a contemporary system of psychodiagnostics by clinical phenomenology, the third edition of the DSM replaced hysterical neurosis with dissociative disorders and conversion disorders under the broader classification of somatoform disorders [62]. Early hypotheses regarding the etiology of hysteria were carried forward into thinking about somatoform disorders. Psychoanalytic theories suggested the repressed expression of conflicted unconscious drives, learning theories held that people with conversion disorders benefitted from secondary gain of their somatic symptoms, and sociocultural hypotheses were that somatic symptoms occur in substitution of the expression of intense forbidden ideas and emotions [63].

In 2013, the term ‘functional neurological symptom disorder’ was introduced in the DSM Version 5 Text Revision (DSM-V-TR) [64] and conversion disorder remained the main nomenclature. The 2022 revision of DSM-V-TR then changed the primary name to functional neurological symptom disorder and maintained conversion disorder as a synonym (Box 1) [65]. FND is now classified by the International Classification of Diseases (ICD-11) [59] as a dissociative neurological symptom disorder, defined as a mental health condition involving a loss of connection between thoughts, memories, feelings, surroundings, behavior, and identity [66]. More recent data from neuroscientific studies [67,68,69,70,71,72] have been used to support claims of emotional processing that might be familiar to earlier advocates of hysteria and somatoform conditions. Despite poor-quality supporting research [73], mainstay interventions for FND continue to include psychodynamic and cognitive-behavior therapies to address emotional processing. Thus, despite the original intent [74] and subsequent rationalizations [75] of the principal proponents of FND, this brief historical analysis indicates a continuous underlying conceptual thread that remains unbroken between neurasthenia, through hysterical neurosis and somatoform disorders, leading to the contemporary psychosomatic/sociogenic illness construction of FND.

Box 1 **Diagnostic criteria for functional neurologic disorder [61]**

- One or more symptoms of altered voluntary motor behavior or sensory function
- Clinical findings provide evidence of incompatibility between the symptom and recognized neurological or medical conditions
- The symptom or deficit is not better explained by another medical or mental disorder
- The symptom or deficit causes clinically significant distress or impairment in social, occupational, or other important areas of functioning, or warrants medical evaluation

Proponents suggest that just the label of FND may be helpful for some patients who live with troublesome but medically unexplained symptoms and signs [76,77]. Even still, the DSM-V-TR diagnostic criteria for FND indicate this label should not be provided when an alternative diagnosis is more compelling. For example, ME/CFS should not be considered as a functional disorder because more specific case definition criteria best explain its constellation of symptoms, signs, and pathophysiology. However, some prominent medical organizations have conflated PEM/PENE with FND because some symptoms of PEM/PENE are represented among the DSM-V-TR case definition criteria for FND. For example, the UK NICE attempted to classify ME/CFS as a functional disorder in 2017 [78]. This action was met with significant opposition from the ME community [79]. The dispute lasted over two years with the subsequent guideline removing the reference to ME/CFS as FND [78]. Both individual clinicians (generally from the fields of neurology and psychiatry) and prominent national medical organizations in European countries [80,81] persist in classifying ME/CFS as FND despite compelling evidence to the contrary.

## 4. Evidence Refutes That Long COVID Should Be Considered a Functional Neurologic Disorder

FND refers to medical and neurologic symptoms that fail to match any existing medical or neurological conditions [82]. It is a rare syndrome, affecting around 4–12 per 100,000 people despite a suggestion that it is commonly diagnosed in neurology clinics [83]. FND is usually diagnosed when patients are observed to experience seizure-like spells in the setting of normal electroencephalography (EEG) or when they demonstrate abnormal movements or paralysis that are incongruent with their neurologic exam and neuroimaging. In some patients, FND may occur alongside other diagnosable entities, such as long COVID. In this scenario, the clinician should evaluate and treat those diagnosable entities, and refrain from considering the entire patient presentation as “functional” simply because functional aspects may be present. While the rate of misdiagnosis with FND in patients with long COVID is unknown, clinical experience suggests that many patients with complex chronic disorders in general have been misdiagnosed with anxiety, depression, or FND at some point in the course of their illness (Box 2).

Box 2 **Defined conditions commonly misdiagnosed as functional neurologic disorder**

- **Autonomic conditions**, such as neurocardiogenic syncope, postural orthostatic tachycardia syndrome, orthostatic intolerance, and autonomic and small fiber neuropathy
- **Chronic pain conditions**, such as fibromyalgia, myofascial pain syndrome, and complex regional pain syndrome
- **Systemic immune conditions**, such as mast cell activation syndrome and mastocytosis
- **Autoimmune conditions**, such as Sjögren’s syndrome, systemic lupus erythematosus, and anti-phospholipid syndrome
- **Genetic conditions**, such as hypermobile Ehlers-Danlos syndrome and other hypermobility spectrum disorders, Fabry’s disease and others
- **Mitochondrial and metabolic conditions**
- **Infection-associated chronic conditions**, such as myalgic encephalomyelitis, Long COVID/Post-COVID condition, and post-treatment Lyme disease

### 4.1. Refutative Evidence from Pathophysiology

The scope and severity of Long COVID-related disablement in individuals and in society has incentivized investigations into the pathophysiology of this novel infection-associated chronic disease. Long COVID is now understood as an umbrella term encompassing a complex pathophysiology affecting multiple organ systems. Various potential aspects of long COVID pathobiology include autonomic manifestations [84]; vascular and endothelial dysfunction in the context of hypercoagulability [85,86,87]; viral persistence [88]; abnormalities in T cell populations and responses [89,90]; impaired cardiopulmonary function [91,92]; autoimmunity [93,94,95]; bioenergetic impairments [96,97,98]; small fiber neuropathy [99]; and alterations in the gut microbiome [100]. In 2021, post-COVID-19 condition (or, long COVID) was assigned an ICD code (U09.9) [101]. The collective understanding of long COVID is far from settled. However, an accumulating science now provides a more compelling pathophysiological basis for testing and interventions than considering long COVID as a functional disorder. Long COVID should not be broadly considered as FND because of its “organic” nature, requiring FND to be ruled out according to DSM-V-TR criteria [64].

### 4.2. Refutative Evidence from Clinical Presentation

Typically, people with functional disorders often exhibit numerous multi-systemic and multi-organ concerns, including various neurologic and psychiatric manifestations such as sensory disturbance, motor weakness, balance difficulty, chronic dizziness, chronic vertigo, chronic pain, chronic fatigue, sleep impairment, urinary and gastrointestinal symptoms, and cognitive dysfunction (Table 1). The symptom experience and distress associated with symptoms in people with FND is frequently not supported or incongruent with objective findings on neurologic examination and diagnostic testing. People with functional disorders also may have comorbid psychiatric conditions, such as depression and anxiety [102,103,104,105]. It remains unclear whether the prevalence and severity of psychiatric comorbidities among those with functional disorders is greater than people living with other types of chronic illnesses, and whether psychiatric conditions contributed to other signs and symptoms or are a secondary reaction to their presence. In addition, inventories used to measure anxiety and depression often may capture the autonomic signs and symptoms of underlying pathobiological process. FND, if diagnosed correctly through positive signs on neurologic examination, does not appear to be as common although true prevalence is unknown and needs to be studied. Moreover, no carefully designed research studies have been conducted to systematically test the hypothesis that long COVID is a functional disorder and the extant literature is poor in methodological quality [106].

#### 4.2.1. Motor Examination

The diagnosis of FND requires the presence of discrete neurologic deficits, which are usually elicited as part of the neurologic examination (Table 1 and Table 2). Presenting features may include weakness in the lower or upper extremities of sudden onset and can be unilateral or bilateral. A neurologic examination is used to demonstrate evidence of internal inconsistency between voluntary movements and automatic movements through findings of a positive Hoover’s sign and hip abductor sign [107]. While weakness in the extremities is a common concern in many people living with long COVID, clinical experience indicates that the neurologic examination typically reveals an unremarkable motor examination without Hoover’s or hip abductor signs. Give-way weakness may be present in patients with long COVID, but it is usually diffuse and non-lateralizing and occurs secondary to pain, fatigue, PEM/PENE, or orthostatic intolerance. In the context of these companion findings, give-way weakness should not be interpreted as evidence of a functional etiology.

**Table 2:** Key differentiating physical examination findings of long COVID vs. functional neurologic disorder.

Findings	Long COVID	Functional Neurological Disorder
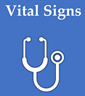	Postural tachycardia Orthostatic hypotension Dizziness Other symptoms upon standing that are relieved by sitting or lying down	No usual abnormalities
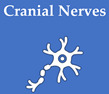	May have dilated poorly reactive pupils or mild horizontal end-point nystagmus	Normal, although patients may report vision or hearing impairment
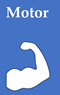	Give-way weakness may be present due to fatigue, post-exertional neuroimmune exhaustion, misunderstanding the task, or poor effort	Weakness inconsistent with known neurologic patterns Paralysis and weakness with positive Hoover’s sign and/or positive hip abductor sign
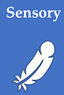	Length and non-length dependent reduced temperature and pinprick consistent with small fiber neuropathy	Complete anesthesia in certain body parts or exactly at the midline or below the waist, incongruent with known neurologic patterns
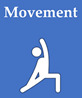	Whole-body shaking, mild postural tremor, and/or internal tremor not visible to the examiner Possible fasciculations due to benign fasciculation syndrome	Tremor entrainment Tremor that disappears with distraction Inconsistent tremor Unusual tremor incongruent with neurologic disorders
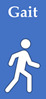	Usually normal, but some unsteadiness and difficulty with tandem walking might be present	Functional gait Astasia-abasia Unusual gait pattern inconsistent with another neurological cause
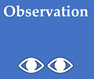	Syncope Presyncope Orthostatic intolerance Anoxic seizures	Spells with non-epileptic convulsions
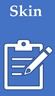	Acrocyanosis with discoloration of the legs and/or arms distally, more in the dependent position (Figure 2) Possible dermatographia Possible dry skin Possible pale or flushed appearance Possible maculopapular rashes, urticarial lesions, and chilblains	No usual abnormalities

#### 4.2.2. Sensory Examination

A common presenting feature of FND is sensory disturbance that fails to fit into defined patterns of neuropathy, radiculopathy, or the central lesion of the brain (Table 1 and Table 2). Sensory testing as part of neurologic examination in a patient with FND may reveal complete anesthesia in non-anatomic distributions, such as involving an entire extremity, located exactly at the midline, or below the waist. While sensory disturbance is common in patients with long COVID due, in part, to post-COVID-19 small fiber neuropathy [108], sensory exam findings usually correspond to a neuropathic pattern with decreased pinprick and temperature sensations in the feet or hands, distally more than proximally. However, a patchy sensory loss distribution is not uncommon in those who present with non-length-dependent patchy small fiber neuropathy, a form that is especially prevalent in people with autoimmune disorders [109].

#### 4.2.3. Tremor

Tremor may be another presenting feature of FND with examination findings revealing tremor entrainment and other inconsistent tremor characteristics (Table 1 and Table 2). Tremor and other abnormal movements may be among common complaints of patients with long COVID, but often involve diffuse, whole-body body tremors or shaking, which may be associated with dysautonomia and hyperadrenergic state, including abnormal blood pressure, heart rate, and blood volume. Autonomic dysfunction affects nearly 70% of patients with long COVID [110], so improvement or resolution of abnormal movements associated with dysautonomia may be noted with hydration, increased salt intake, or medications. Additionally, the sensation of “internal vibrations” is often described by people living with long COVID; although the etiology of this concern is not fully understood, clinical experience suggests it often occurs in patients with hypovolemia, dysautonomia, and small fiber neuropathy. These features should not be attributed to functional causes or FND.

#### 4.2.4. Spells and Seizures

Spells of unknown etiology are often attributed to FND, especially when accompanied by normal EEG in the setting of convulsive activity (Table 1). While a small subset of people living with long COVID could have non-epileptic functional seizures, many patients with long COVID have post-COVID-19 dysautonomia in the form of neurocardiogenic syncope, postural orthostatic tachycardia syndrome, orthostatic hypotension, and orthostatic intolerance. These patients often experience spells of presyncope or syncope, some with convulsive activity during syncope, which is termed anoxic seizures. Patients with presyncope and syncope may be misdiagnosed with FND by neurologists with limited knowledge of the phenomenology of syncope. In cases where the etiology of spells is unclear, a tilt table test can provide differentiation between syncope and pseudo-syncope with adequate sensitivity and specificity [111]. A video recording of the spells obtained by the family also may be reviewed by a neurologist to assist with differentiating between syncope and dissociative/functional seizures.

#### 4.2.5. Gait Examination

Gait examination of people living with long COVID is typically unremarkable, although some people may have unsteadiness due to orthostasis or poor proprioception related to large fiber neuropathy, chronic vestibulopathy, or hypermobility spectrum disorders (Table 1 and Table 2). Clinical features of abnormal gait and movements consistent with FND, such as dystonia, ticks, twitches, and jerks, are typically uncommon in people with long COVID. Concerns about gait and findings in the gait examination explainable by other causes should not be taken as signs of FND.

#### 4.2.6. Urinary Functioning

Urinary retention is sometimes listed as a feature of FND [112], but clinical experience suggests that urinary retention rarely occurs in people with long COVID. Urinary retention is a common feature of autonomic neuropathy and, if present in patients with long COVID, should prompt an investigation for post-COVID-19 autonomic neuropathy or ganglionopathy which have been described as rare post-COVID-19 conditions [113].

#### 4.2.7. Cognition

Cognitive concerns, such as difficulty with attention, concentration, and memory, have been endorsed by some proponents of FND as being functional in nature. One review suggested that almost one quarter of patients attending memory clinics may have functional cognitive disorders [114]. However, it is unclear how these complaints are differentiated from the cognitive impairment—commonly referred to as brain fog—in patients with long COVID. Numerous studies suggested neuroinflammation and microglial activation as mechanisms of post-COVID-19 neurologic sequelae [115,116]. Importantly, several studies identified neuropsychological deficits via cognitive testing in patients with long COVID [117,118].

Moreover, traditional screening tests for evaluation of cognitive impairment designed to screen patients for Alzheimer’s disease are not useful in patients with cognitive impairment secondary to long COVID [119]. Thus, currently available screening tests that were designed for neurodegenerative conditions and not neuroinflammatory or neuroimmune conditions are inadequate to rule in or out impairments secondary to neuroinflammatory and neuroimmune processes, and, therefore, cannot be utilized to diagnose as functional by default if results are “normal” in patients with long COVID and other disorders that are associated with cognitive complaints. Cognitive tests to assess patients with non-neurodegenerative cognitive complaints need to be designed to provide clinicians with validated tools to better evaluate and quantify the extent of cognitive impairment in patients with post-COVID-19 neurocognitive syndrome.

#### 4.2.8. Summary

**Figure 2:**
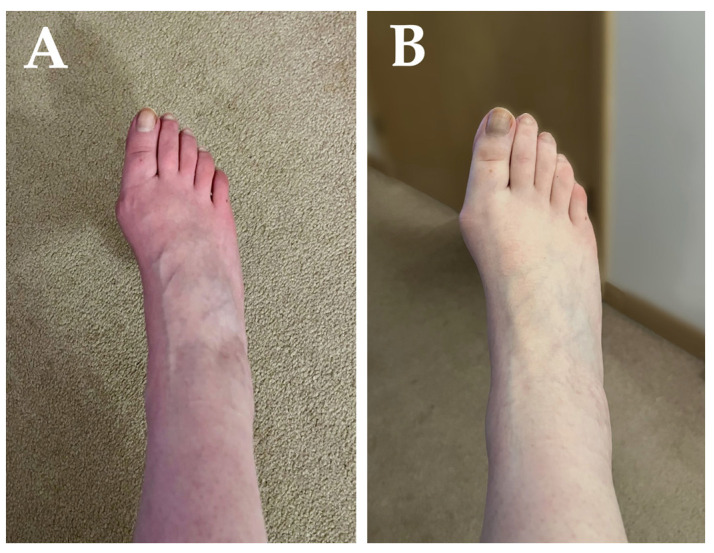
Acrocyanosis in the distal leg in the dependent position (**A**) that immediately disappears on raising the leg up against gravity (**B**) in a person with long COVID. She was initially misdiagnosed with functional neurologic disorder based on non-epileptic spells, which were subsequently determined to be pre-syncopal episodes caused by post-COVID-19 postural orthostatic tachycardia syndrome.

In summary, FND and long COVID can be effectively differentiated through a comprehensive clinical examination. One caveat is the difficulty that arises when a patient presents with some evidence of functional neurologic disorder on physical examination (e.g., with tremor entrainment or positive Hoover’s sign) in conjunction with postural tachycardia, acrocyanosis, and other features of long COVID and post-COVID-19 dysautonomia or small fiber neuropathy (Figure 2). In cases like these, management of non-FND disorders and symptoms should be the top priority. Clinical experience suggests that a significant number of patients with long COVID are being misdiagnosed with FND without diagnosing and addressing the primary long COVID pathophysiology or symptoms, such as dizziness, palpitations, tachycardia, and pain. In these cases, FND-tailored diagnostic and therapeutic approaches delay improvement and recovery by failing to implement the pharmacologic and non-pharmacologic therapies targeting underlying autonomic, neuropathic, and cardiovascular pathophysiologies. Referral to FND-tailored rehabilitation programs should be considered only for patients, in whom post-COVID-19 FND is determined to be the main component of long COVID and in strict adherence with relevant case definition criteria [82].

### 4.3. Refutative Evidence from Neuroimaging

Despite its relatively recent recognition, the literature describing significant structural brain abnormalities in long COVID is already extensive. This literature suggests FND cannot commonly explain long COVID, because it indicates neurologic signs, symptoms, and disability may be caused by structural changes in the brain. Such abnormalities have been demonstrated using various imaging modalities and range from changes in gray matter thickness and volume, to macro- and microstructural white matter changes and evidence of metabolic and neuroinflammatory derangement. It is also important to note that despite clearly distinctive systemic immunological abnormalities [120,121,122] and abnormal neuroimmune profiles [121,123] in long COVID, routine clinical structural magnetic resonance imaging (MRI) sequences often return normal findings [123].

Douaud et al. [124] examined structural brain changes in a biobank cohort based in the United Kingdom, before and after SARS-CoV-2 infection. Compared to uninfected controls, the authors found significant gray matter thickness reduction following infection, with a reduction in global brain size. Hosp et al. [125] used an MRI diffusion microstructure imaging technique to evaluate subtle changes in both gray and white matter integrity. Compared to recovered infected patients, those with ongoing symptoms demonstrated widespread changes in microstructure, which correlated with evaluations of cognitive dysfunction. Wu et al. [126] used another diffusion tensor imaging (DTI) technique to evaluate the perivascular space and glymphatic system. These authors calculated flow in the perivascular spaces alongside medullary veins, which lie orthogonal to the projection and association nerve fibers in the periventricular deep white matter. They reported reduction in the indices for glymphatic function in people living with long COVID even following a mild acute infection. Another small cohort study compared recovered, brain fog positive, and brain fog negative patients with long COVID [127]. Dynamic contrast-enhanced MRI (DCE-MRI) showed significant whole brain leakage, indicating increased blood–brain barrier (BBB) permeability, in only the ‘brain fog’ sub-group [127]. Chaganti et al. combined techniques in a longitudinal study of 14 patients with long COVID-related cognitive impairment [128]. DCE-MRI and DTI revealed impairments in the integrity of BBB and white matter microstructure [128]. Simultaneously, MR spectroscopy demonstrated reduced glutamate/glutamine in these areas, leading the authors to suggest that white matter injury may result from glutamatergic excitotoxicity, secondary to reduced BBB integrity associated with neuroinflammation [128]. VanElzakker et al. [129] used positron emission tomography (PET) with a tracer for activated microglia ([11C]PBR28) to report evidence of significantly increased neuroinflammation in many brain regions in LC. Peluso et al. [130] used a novel PET tracer ([18F]F-AraG) to tag activated T cells. Following SARS-CoV-2 infection, activated T cells were found in multiple organs including the bowel and bone marrow, but notably had trafficked into central nervous system (CNS) sites such as the brainstem and spinal cord, where they should be absent. This finding was more exaggerated in patients with long COVID signs and symptoms [130]. Biopsy-accessible tissues such as colon tissue demonstrated residual viral components, and the authors speculated they also might be present in the CNS [130].

A 2021 review article canvased the literature of neuroimaging in FND [131]. The highlighted modalities were functional MRI (fMRI), using both resting-state and task-based paradigms; high-resolution structural MRI evaluation of gray matter; DTI of white matter microstructure; MR spectroscopy; CT/MR positron emission tomography; and near-infrared spectroscopy. The authors note that neuroimaging in FND is early in its development, with few replicated studies, and with confounding factors in terms of clinical heterogeneity and co-morbidities. They conclude by encouraging a multimodal neuroimaging approach to advance the field. Most fMRI studies using blood oxygen level-dependent (BOLD) techniques have shown abnormalities in specific brain regions, yet data have been inconsistent [132]. Very recently, Schneider et al. [133] have attempted to further define the variability of BOLD signal in FND, with particular emphasis on the somatomotor, limbic, and salience networks. However, when structural abnormalities have been found in gray [134] or white matter [67], it remains unclear whether they are a cause, consequence, or comorbidity [132,135].

While neuroimaging in FND is an evolving field, there are already replicated findings in long COVID that point toward a coherent structural pathophysiology. Aspects highlighted in the literature to date involve neuroinflammation with microglial activation, a dysfunctional blood–brain barrier, white matter microstructural changes, as well as reduction in gray matter volume. Systemic dysfunction, such as orthostatic intolerance with reduced cerebral blood flow, is also shown to be a key contributor to symptoms [136,137,138,139,140]. The detail of how these findings are driven from specific and potentially correctable upstream causes is enthusiastically anticipated by patients, clinicians, and researchers alike.

## 5. Conclusions

Long COVID continues to be a major public health issue [141]. While several phenotypes of long COVID clinical presentation have emerged based on observational studies and collective clinical experience over the past four years, it is important to emphasize that the vast majority of patients with long COVID do not have FND. As this perspective indicates, long COVID is not based in ‘functional’ etiology, as demonstrated by numerous studies identifying a complex pathophysiology as well as common findings from the clinical examination and a summary of extant structural neuroimaging studies. Further research is needed to delineate precise pathophysiological pathways and effective therapies for long COVID and numerous post-COVID-19 neurologic manifestations. Additionally, studies applying accepted case definition criteria are also needed to determine the true prevalence of FND as the sole or major contributor to symptoms and disability among individuals with persistent symptoms following SARS-CoV-2 infection. These studies will help establish clinical practices that best differentiate this small subset of patients with FND-related specialized needs from the vast majority of people who experience long COVID in the forms of ME/CFS, dysautonomia, immune dysfunction, small fiber neuropathy and other post-COVID-19 neurologic syndromes.

## Figures and Tables

**Table 1:** Typical clinical features of long COVID, myalgic encephalomyelitis/chronic fatigue syndrome, and functional neurologic disorder.

Clinical Feature	ME/CFS	Long COVID	FND
Post-exertional malaise/ Post-exertional neuroimmune exhaustion	Yes	Yes, some types	No
Pain	Yes	Yes, some types	Sometimes
Dizziness	Yes	Yes	No
Neuropathic features	Yes	Yes	No
Recurrent flu-like symptoms	Yes	Common	No
Dysautonomia	Yes	Common	No
Abnormal sleep study	Yes	Yes	No
Fatigue	Yes	Yes	Yes
Impaired sleep	Yes	Yes	Yes
Functional leg weakness	No	No	Yes
Functional seizures	No	No	Yes
Functional tremor	No	No	Yes
Functional dystonia	No	No	Yes
Functional gait disorder	No	No	Yes
Functional facial spasm	No	No	Yes
Functional tics	No	No	Yes
Functional drop attacks	No	No	Yes
Functional sensory symptoms	No	No	Yes
Functional cognitive symptoms	No	No	Yes
Functional speech and swallowing	No	No	Yes
Functional visual symptoms	No	No	Yes
Dissociative symptoms	No	No	Yes

## Data Availability

Not applicable.

## References

[1] Beard G. (1869). Neurasthenia, or nervous exhaustion. Boston Med. Surg. J..

[2] Goetz C.G. (2001). Poor Beard!! Charcot’s internationalization of neurasthenia, the “American disease”. Neurology.

[3] Hickie I., Davenport T., Wakefield D., Vollmer-Conna U., Cameron B., Vernon S.D., Reeves W.C., Lloyd A., Dubbo Infection Outcomes Study Group (2006). Post-infective and chronic fatigue syndromes precipitated by viral and non-viral pathogens: Prospective cohort study. BMJ.

[4] Holmes G.P., Kaplan J.E., Gantz N.M., Komaroff A.L., Schonberger L.B., Straus S.E., Jones J.F., Dubois R.E., Cunningham-Rundles C., Pahwa S. (1988). Chronic fatigue syndrome: A working case definition. Ann. Intern. Med..

[5] Ikuta K., Yamada T., Shimomura T., Kuratsune H., Kawahara R., Ikawa S., Ohnishi E., Sokawa Y., Fukushi H., Hirai K. (2003). Diagnostic evaluation of 2′, 5′-oligoadenylate synthetase activities and antibodies against Epstein-Barr virus and Coxiella burnetii in patients with chronic fatigue syndrome in Japan. Microbes Infect..

[6] Klimas N.G., Salvato F.R., Morgan R., Fletcher M.A. (1990). Immunologic abnormalities in chronic fatigue syndrome. J. Clin. Microbiol..

[7] Kogelnik A.M., Loomis K., Hoegh-Petersen M., Rosso F., Hischier C., Montoya J.G. (2006). Use of valganciclovir in patients with elevated antibody titers against Human Herpesvirus-6 (HHV-6) and Epstein-Barr Virus (EBV) who were experiencing central nervous system dysfunction including long-standing fatigue. J. Clin. Virol..

[8] Lerner A.M., Beqaj S.H., Deeter R.G., Fitzgerald J.T. (2004). IgM serum antibodies to Epstein-Barr virus are uniquely present in a subset of patients with the chronic fatigue syndrome. In Vivo.

[9] Shikova E., Reshkova V., Kumanova A., Raleva S., Alexandrova D., Capo N., Murovska M., On Behalf of the European Network on Me/Cfs Euromene (2020). Cytomegalovirus, Epstein-Barr virus, and human herpesvirus-6 infections in patients with myalgic encephalomyelitis/chronic fatigue syndrome. J. Med. Virol..

[10] Kasimir F., Toomey D., Liu Z., Kaiping A.C., Ariza M.E., Prusty B.K. (2022). Tissue specific signature of HHV-6 infection in ME/CFS. Front. Mol. Biosci..

[11] Sejvar J.J., Curns A.T., Welburg L., Jones J.F., Lundgren L.M., Capuron L., Pape J., Reeves W.C., Campbel G.L. (2008). Neurocognitive and functional outcomes in persons recovering from West Nile virus illness. J. Neuropsychol..

[12] Sneller M.C., Reilly C., Badio M., Bishop R.J., Eghrari A.O., Moses S.J., Johnson K.L., Gayedyu-Dennis D., Hensley L.E., Prevail III Study Group (2019). A longitudinal study of Ebola sequelae in Liberia. N. Engl. J. Med..

[13] Kelly J.D., Van Ryn C., Badio M., Fayiah T., Johnson K., Gayedyu-Dennis D., Weiser S.D., Porco T.C., Martin J.N., Sneller M.C. (2022). Clinical sequelae among individuals with pauci-symptomatic or asymptomatic Ebola virus infection and unrecognised Ebola virus disease in Liberia: A longitudinal cohort study. Lancet Infect. Dis..

[14] Chia J.K. (2005). The role of enterovirus in chronic fatigue syndrome. J. Clin. Pathol..

[15] Chia J.K., Chia A.Y. (2008). Chronic fatigue syndrome is associated with chronic enterovirus infection of the stomach. J. Clin. Pathol..

[16] Ledina D., Bradaric N., Milas I., Ivic I., Brncic N., Kuzmicic N. (2007). Chronic fatigue syndrome after Q fever. Med. Sci. Monit..

[17] Treib J., Grauer M.T., Haass A., Langenbach J., Holzer G., Woessner R. (2000). Chronic fatigue syndrome in patients with Lyme borreliosis. Eur. Neurol..

[18] Fares-Medina S., Diaz-Caro I., Garcia-Montes R., Corral-Liria I., Garcia-Gomez-Heras S. (2022). Multiple Chemical Sensitivity Syndrome: First symptoms and evolution of the clinical picture: Case-control study/epidemiological case-control study. Int. J. Environ. Res. Public Health.

[19] Katerndahl D.A., Bell I.R., Palmer R.F., Miller C.S. (2012). Chemical intolerance in primary care settings: Prevalence, comorbidity, and outcomes. Ann. Fam. Med..

[20] National Academies of Sciences, Engineering, and Medicine (2024). A Long COVID Definition: A Chronic, Systemic Disease State with Profound Consequences.

[21] Chen C., Haupert S.R., Zimmermann L., Shi X., Fritsche L.G., Mukherjee B. (2022). Global Prevalence of Post COVID-19 Condition or long COVID: A meta-analysis and systematic review. J. Infect. Dis..

[22] Xie Y., Xu E., Bowe B., Al-Aly Z. (2022). Long-term cardiovascular outcomes of COVID-19. Nat. Med..

[23] Bowe B., Xie Y., Al-Aly Z. (2023). Postacute sequelae of COVID-19 at 2 years. Nat. Med..

[24] Cai M., Xie Y., Topol E.J., Al-Aly Z. (2024). Three-year outcomes of post-acute sequelae of COVID-19. Nat. Med..

[25] Davis H.E., Assaf G.S., McCorkell L., Wei H., Low R.J., Re’em Y., Redfield S., Austin J.P., Akrami A. (2021). Characterizing long COVID in an international cohort: 7 months of symptoms and their impact. eClinicalMedicine.

[26] Bai N.A., Richardson C.S. (2023). Posttreatment Lyme disease syndrome and myalgic encephalomyelitis/chronic fatigue syndrome: A systematic review and comparison of pathogenesis. Chronic Dis. Transl. Med..

[27] Ruiz-Pablos M., Paiva B., Montero-Mateo R., Garcia N., Zabaleta A. (2021). Epstein-Barr Virus and the origin of myalgic encephalomyelitis or chronic fatigue syndrome. Front. Immunol..

[28] Jason L.A., Katz B.Z., Shiraishi Y., Mears C.J., Im Y., Taylor R. (2014). Predictors of post-infectious chronic fatigue syndrome in adolescents. Health Psychol. Behav. Med..

[29] Jason L.A., Dorri J.A. (2022). ME/CFS and post-exertional malaise among patients with long COVID. Neurol. Int..

[30] Twomey R., DeMars J., Franklin K., Culos-Reed S.N., Weatherald J., Wrightson J.G. (2022). Chronic fatigue and postexertional malaise in people living with long COVID: An observational study. Phys. Ther..

[31] Vernon S.D., Hartle M., Sullivan K., Bell J., Abbaszadeh S., Unutmaz D., Bateman L. (2023). Post-exertional malaise among people with long COVID compared to myalgic encephalomyelitis/chronic fatigue syndrome (ME/CFS). Work.

[32] Jason L.A., Islam M., Conroy K., Cotler J., Torres C., Johnson M., Mabie B. (2021). COVID-19 symptoms over time: Comparing long-haulers to ME/CFS. Fatigue.

[33] Tokumasu K., Honda H., Sunada N., Sakurada Y., Matsuda Y., Yamamoto K., Nakano Y., Hasegawa T., Yamamoto Y., Otsuka Y. (2022). Clinical characteristics of myalgic encephalomyelitis/chronic fatigue syndrome (ME/CFS) diagnosed in patients with long COVID. Medicina.

[34] Little J., Higgins M., Palepu R. (2024). Long COVID—Can we deny a diagnosis without denying a person’s reality?. Australas. Psychiatry.

[35] Leitch A.G. (1995). Neurasthenia, myalgic encephalitis or cryptogenic chronic fatigue syndrome?. QJM.

[36] Gilliam A.G. (1934). Epidemiological Study on an Epidemic, Diagnosed as Poliomyelitis, Occurring among the Personnel of Los Angeles County General Hospital during the Summer of 1934.

[37] Sigurdsson B., Sigurjonsson J., Sigurdsson J.H., Thorkelsson J., Gudmundsson K.R. (1950). A disease epidemic in Iceland simulating poliomyelitis. Am. J. Hyg..

[38] Ramsay A.M. (1981). Myalgic encephalomyelitis: A baffling syndrome with a tragic aftermath. ME Assoc. J..

[39] Ramsay A.M. (1986). Myalgic Encephalomyelitis and Postviral Fatigue States: The Saga of Royal Free Disease.

[40] (1978). Epidemic myalgic encephalomyelitis. Br. Med. J..

[41] Evengard B., Schacterle R.S., Komaroff A.L. (1999). Chronic fatigue syndrome: New insights and old ignorance. J. Intern. Med..

[42] Jason L.A., Eisele H., Taylor R.R. (2001). Assessing attitudes toward new names for chronic fatigue syndrome. Eval. Health Prof..

[43] Carruthers B.M., Jain A.K., DeMeirleir K.L., Peterson D.L., Klimas N.G., Lerner A.M., Flor-Henry P., Joshi P., Powles A.C., Sherkey J.A. (2003). Myalgic encephalomyelitis/chronic fatigue syndrome: Clinical working case definition, diagnostic and treatment protocols. J. Chronic Fatigue Syndr..

[44] Carruthers B.M., van de Sande M.I., De Meirleir K.L., Klimas N.G., Broderick G., Mitchell T., Staines D., Powles A.C., Speight N., Vallings R. (2011). Myalgic encephalomyelitis: International Consensus Criteria. J. Intern. Med..

[45] Chu L., Valencia I.J., Garvert D.W., Montoya J.G. (2018). Deconstructing post-exertional malaise in myalgic encephalomyelitis/chronic fatigue syndrome: A patient-centered, cross-sectional survey. PLoS ONE.

[46] Chu L., Valencia I.J., Garvert D.W., Montoya J.G. (2019). Onset patterns and course of myalgic encephalomyelitis/chronic fatigue syndrome. Front. Pediatr..

[47] Fukuda K., Straus S.E., Hickie I., Sharpe M.C., Dobbins J.G., Komaroff A. (1994). The chronic fatigue syndrome: A comprehensive approach to its definition and study. International Chronic Fatigue Syndrome Study Group. Ann. Intern. Med..

[48] Jason L.A., Torres-Harding S.R., Carrico A.W., Taylor R.R. (2002). Symptom occurrence in persons with chronic fatigue syndrome. Biol. Psychol..

[49] Stussman B., Williams A., Snow J., Gavin A., Scott R., Nath A., Walitt B. (2020). Characterization of post-exertional malaise in patients with myalgic encephalomyelitis/chronic fatigue syndrome. Front. Neurol..

[50] Van Ness J.M., Stevens S.R., Bateman L., Stiles T.L., Snell C.R. (2010). Postexertional malaise in women with chronic fatigue syndrome. J. Womens Health.

[51] O’Brien K.K., Brown D.A., Bergin C., Erlandson K.M., Vera J.H., Avery L., Carusone S.C., Cheung A.M., Goulding S., Harding R. (2022). Long COVID and episodic disability: Advancing the conceptualisation, measurement and knowledge of episodic disability among people living with Long COVID—Protocol for a mixed-methods study. BMJ Open.

[52] O’Brien K.K., Brown D.A., McDuff K., St Clair-Sullivan N., Solomon P., Chan Carusone S., McCorkell L., Wei H., Goulding S., O’Hara M. (2023). Conceptualising the episodic nature of disability among adults living with Long COVID: A qualitative study. BMJ Glob. Health.

[53] Sharpe M.C., Archard L.C., Banatvala J.E., Borysiewicz L.K., Clare A.W., David A., Edwards R.H., Hawton K.E., Lambert H.P., Lane R.J. (1991). A report–chronic fatigue syndrome: Guidelines for research. J. R. Soc. Med..

[54] Reeves W.C., Wagner D., Nisenbaum R., Jones J.F., Gurbaxani B., Solomon L., Papanicolaou D.A., Unger E.R., Vernon S.D., Heim C. (2005). Chronic fatigue syndrome—A clinically empirical approach to its definition and study. BMC Med..

[55] United States National Academy of Medicine (2015). Beyond Myalgic Encephalomyelitis/Chronic Fatigue Syndrome: Redefining an Illness. The National Academies Collection: Reports Funded by National Institutes of Health.

[56] National Institute for Health and Care Excellence (2021). Myalgic Encephalomyelitis (or Encephalopathy)/Chronic Fatigue Syndrome: Diagnosis and Management—NICE Guideline, No. 206.

[57] (1956). A new clinical entity?. Lancet.

[58] World Health Organization (1969). Manual of the International Statistical Classification of Diseases, Injuries, and Causes of Death Based on the Recommendations of the Eighth Revision Conference.

[59] World Health Organization (2022). ICD-11: International Classification of Diseases, 11th revision.

[60] American Psychiatric Association (1952). Committee on Nomenclature and Statistics. Mental Disorders.

[61] American Psychiatric Association (1968). Committee on Nomenclature and Statistics. Diagnostic and Statistical Manual of Mental Disorders.

[62] American Psychiatric Association (1980). Diagnostic and Statistical Manual of Mental Disorders.

[63] Owens C., Dein S. (2006). Conversion disorder: The modern hysteria. Adv. Psychiatr. Treat..

[64] American Psychiatric Association (2013). Diagnostic and Statistical Manual of Mental Disorders.

[65] American Psychiatric Association (2022). Diagnostic and Statistical Manual of Mental Disorders: DSM-5-TR.

[66] Mayo Clinic Dissociative Disorders. https://www.mayoclinic.org/diseases-conditions/dissociative-disorders/symptoms-causes/syc-20355215.

[67] Diez I., Williams B., Kubicki M.R., Makris N., Perez D.L. (2021). Reduced limbic microstructural integrity in functional neurological disorder. Psychol. Med..

[68] Hassa T., Spiteri S., Schmidt R., Merkel C., Schoenfeld M.A. (2021). Increased amygdala activity associated with cognitive reappraisal strategy in functional neurologic disorder. Front. Psychiatry.

[69] Ospina J.P., Jalilianhasanpour R., Perez D.L. (2019). The role of the anterior and midcingulate cortex in the neurobiology of functional neurologic disorder. Handb. Clin. Neurol..

[70] Perez D.L., Matin N., Williams B., Tanev K., Makris N., LaFrance W.C., Dickerson B.C. (2018). Cortical thickness alterations linked to somatoform and psychological dissociation in functional neurological disorders. Hum. Brain Mapp..

[71] Perez D.L., Williams B., Matin N., LaFrance W.C., Costumero-Ramos V., Fricchione G.L., Sepulcre J., Keshavan M.S., Dickerson B.C. (2017). Corticolimbic structural alterations linked to health status and trait anxiety in functional neurological disorder. J. Neurol. Neurosurg. Psychiatry.

[72] Williams B., Jalilianhasanpour R., Matin N., Fricchione G.L., Sepulcre J., Keshavan M.S., LaFrance W.C., Dickerson B.C., Perez D.L. (2018). Individual differences in corticolimbic structural profiles linked to insecure attachment and coping styles in motor functional neurological disorders. J. Psychiatr. Res..

[73] Gutkin M., McLean L., Brown R., Kanaan R.A. (2020). Systematic review of psychotherapy for adults with functional neurological disorder. J. Neurol. Neurosurg. Psychiatry.

[74] Stone J., LaFrance W.C., Levenson J.L., Sharpe M. (2010). Issues for DSM-5: Conversion disorder. Am. J. Psychiatry.

[75] Stone J., Hoeritzauer I., McWhirter L., Carson A. (2024). Functional neurological disorder: Defying dualism. World Psychiatry.

[76] McLoughlin C., Hoeritzauer I., Cabreira V., Aybek S., Adams C., Alty J., Ball H.A., Baker J., Bullock K., Burness C. (2023). Functional neurological disorder is a feminist issue. J. Neurol. Neurosurg. Psychiatry.

[77] Stone J., Carson A., Sharpe M. (2005). Functional symptoms and signs in neurology: Assessment and diagnosis. J. Neurol. Neurosurg. Psychiatry.

[78] National Institute for Health and Care Excellence Suspected Neurological Conditions: Recognition and Referral|NICE Guideline [NG127]. https://www.nice.org.uk/guidance/ng127.

[79] ME Association ME Association Petition: M.E. is Not a Functional Disorder. 27 September 2017. https://meassociation.org.uk/2017/09/me-association-petition-m-e-is-not-a-functional-disorder-27-september-2017/.

[80] Anonymous The On-Call Doctor Rejected Line’s Desperate Call Just Days before Her Husband’s Death: ‘He Was Let Down by the System’. https://www-bt-dk.translate.goog/samfund/vagtlaegen-afviste-lines-desperate-opkald-faa-dage-foer-sin-mands-doed-han-blev?_x_tr_sl=da&_x_tr_tl=en&_x_tr_hl=en-US&_x_tr_pto=wapp.

[81] ME Action Advocacy Update from Finland. https://www.meaction.net/2020/12/07/advocacy-update-from-finland/.

[82] American Psychiatric Association (2022). Desk Reference to the Diagnostic Criteria from DSM-5-TR.

[83] Mishra A., Pandey S. (2022). Functional neurological disorders: Clinical spectrum, diagnosis, and treatment. Neurologist.

[84] Hira R., Karalasingham K., Baker J.R., Raj S.R. (2023). Autonomic manifestations of long-COVID syndrome. Curr. Neurol. Neurosci. Rep..

[85] Turner S., Khan M.A., Putrino D., Woodcock A., Kell D.B., Pretorius E. (2023). Long COVID: Pathophysiological factors and abnormalities of coagulation. Trends Endocrinol. Metab..

[86] Boccatonda A., Campello E., Simion C., Simioni P. (2023). Long-term hypercoagulability, endotheliopathy and inflammation following acute SARS-CoV-2 infection. Expert Rev. Hematol..

[87] Smadja D.M., Mentzer S.J., Fontenay M., Laffan M.A., Ackermann M., Helms J., Jonigk D., Chocron R., Pier G.B., Gendron N. (2021). COVID-19 is a systemic vascular hemopathy: Insight for mechanistic and clinical aspects. Angiogenesis.

[88] Proal A.D., VanElzakker M.B., Aleman S., Bach K., Boribong B.P., Buggert M., Cherry S., Chertow D.S., Davies H.E., Dupont C.L. (2023). SARS-CoV-2 reservoir in post-acute sequelae of COVID-19 (PASC). Nat. Immunol..

[89] Yin K., Peluso M.J., Luo X., Thomas R., Shin M.G., Neidleman J., Andrew A., Young K.C., Ma T., Hoh R. (2024). Long COVID manifests with T cell dysregulation, inflammation and an uncoordinated adaptive immune response to SARS-CoV-2. Nat. Immunol..

[90] Roe K. (2021). A role for T-cell exhaustion in Long COVID-19 and severe outcomes for several categories of COVID-19 patients. J. Neurosci. Res..

[91] Durstenfeld M.S., Peluso M.J., Kaveti P., Hill C., Li D., Sander E., Swaminathan S., Arechiga V.M., Lu S., Goldberg S.A. (2023). Reduced exercise capacity, chronotropic incompetence, and early systemic inflammation in cardiopulmonary phenotype long coronavirus disease 2019. J. Infect. Dis..

[92] Durstenfeld M.S., Sun K., Tahir P., Peluso M.J., Deeks S.G., Aras M.A., Grandis D.J., Long C.S., Beatty A., Hsue P.Y. (2022). Use of cardiopulmonary exercise testing to evaluate long COVID-19 symptoms in adults: A systematic review and meta-analysis. JAMA Netw. Open.

[93] Ortona E., Buonsenso D., Carfi A., Malorni W., Long COVID Kids study group (2021). Long COVID: An estrogen-associated autoimmune disease?. Cell Death Discov..

[94] Amiral J., Seghatchian J. (2023). Autoimmune complications of COVID-19 and potential consequences for long-lasting disease syndromes. Transfus. Apher. Sci..

[95] Vojdani A., Vojdani E., Saidara E., Maes M. (2023). Persistent SARS-CoV-2 Infection, EBV, HHV-6 and other factors may contribute to inflammation and autoimmunity in long COVID. Viruses.

[96] Kahn P.A., Joseph P., Heerdt P.M., Singh I. (2024). Differential cardiopulmonary haemodynamic phenotypes in PASC-related exercise intolerance. ERJ Open Res..

[97] Singh I., Joseph P., Heerdt P.M., Cullinan M., Lutchmansingh D.D., Gulati M., Possick J.D., Systrom D.M., Waxman A.B. (2022). Persistent exertional intolerance after COVID-19: Insights from invasive cardiopulmonary exercise testing. Chest.

[98] Appelman B., Charlton B.T., Goulding R.P., Kerkhoff T.J., Breedveld E.A., Noort W., Offringa C., Bloemers F.W., van Weeghel M., Schomakers B.V. (2024). Muscle abnormalities worsen after post-exertional malaise in long COVID. Nat. Commun..

[99] McAlpine L., Zubair A.S., Joseph P., Spudich S. (2024). Case-control study of individuals with small fiber neuropathy after COVID-19. Neurol. Neuroimmunol. Neuroinflamm..

[100] Nagata N., Takeuchi T., Masuoka H., Aoki R., Ishikane M., Iwamoto N., Sugiyama M., Suda W., Nakanishi Y., Terada-Hirashima J. (2023). Human gut microbiota and its metabolites impact immune responses in COVID-19 and its complications. Gastroenterology.

[101] United States Centers for Disease Control and Prevention New ICD-10-CM Code for Post-COVID Conditions, Following the 2019 Novel Coronavirus (COVID-19). https://www.cdc.gov/nchs/data/icd/announcement-new-icd-code-for-post-covid-condition-april-2022-final.pdf.

[102] Novakova L., Anyz J., Forejtova Z., Rosikova T., Vechetova G., Sojka P., Ruzicka E., Serranova T. (2023). Increased frequency of self-reported obsessive-compulsive symptoms in patients with functional movement disorders. Mov. Disord. Clin. Pract..

[103] Roivainen E., Peura M., Patsi J. (2023). Cognitive profile in functional disorders. Cogn. Neuropsychiatry.

[104] Hamilton J., Campos R., Creed F. (1996). Anxiety, depression and management of medically unexplained symptoms in medical clinics. J. R. Coll. Physicians Lond..

[105] Afari N., Ahumada S.M., Wright L.J., Mostoufi S., Golnari G., Reis V., Cuneo J.G. (2014). Psychological trauma and functional somatic syndromes: A systematic review and meta-analysis. Psychosom. Med..

[106] Teodoro T., Chen J., Gelauff J., Edwards M.J. (2023). Functional neurological disorder in people with long COVID: A systematic review. Eur. J. Neurol..

[107] Bennett K., Diamond C., Hoeritzauer I., Gardiner P., McWhirter L., Carson A., Stone J. (2021). A practical review of functional neurological disorder (FND) for the general physician. Clin. Med..

[108] Oaklander A.L., Mills A.J., Kelley M., Toran L.S., Smith B., Dalakas M.C., Nath A. (2022). Peripheral neuropathy evaluations of patients with prolonged long COVID. Neurol. Neuroimmunol. Neuroinflamm..

[109] Gemignani F., Bellanova M.F., Saccani E., Pavesi G. (2022). Non-length-dependent small fiber neuropathy: Not a matter of stockings and gloves. Muscle Nerve.

[110] Larsen N.W., Stiles L.E., Shaik R., Schneider L., Muppidi S., Tsui C.T., Geng L.N., Bonilla H., Miglis M.G. (2022). Characterization of autonomic symptom burden in long COVID: A global survey of 2314 adults. Front. Neurol..

[111] Thijs R.D., Brignole M., Falup-Pecurariu C., Fanciulli A., Freeman R., Guaraldi P., Jordan J., Habek M., Hilz M., Traon A.P. (2021). Recommendations for tilt table testing and other provocative cardiovascular autonomic tests in conditions that may cause transient loss of consciousness: Consensus statement of the European Federation of Autonomic Societies (EFAS) endorsed by the American Autonomic Society (AAS) and the European Academy of Neurology (EAN). Clin. Auton. Res..

[112] Panicker J.N., Selai C., Herve F., Rademakers K., Dmochowski R., Tarcan T., von Gontard A., Vrijens D. (2020). Psychological comorbidities and functional neurological disorders in women with idiopathic urinary retention: International Consultation on Incontinence Research Society (ICI-RS) 2019. Neurourol. Urodyn..

[113] Shouman K., Vanichkachorn G., Cheshire W.P., Suarez M.D., Shelly S., Lamotte G.J., Sandroni P., Benarroch E.E., Berini S.E., Cutsforth-Gregory J.K. (2021). Autonomic dysfunction following COVID-19 infection: An early experience. Clin. Auton. Res..

[114] McWhirter L., Ritchie C., Stone J., Carson A. (2020). Functional cognitive disorders: A systematic review. Lancet Psychiatry.

[115] Monje M., Iwasaki A. (2022). The neurobiology of long COVID. Neuron.

[116] Soung A.L., Vanderheiden A., Nordvig A.S., Sissoko C.A., Canoll P., Mariani M.B., Jiang X., Bricker T., Rosoklija G.B., Arango V. (2022). COVID-19 induces CNS cytokine expression and loss of hippocampal neurogenesis. Brain.

[117] Voruz P., Allali G., Benzakour L., Nuber-Champier A., Thomasson M., Jacot de Alcântara I., Pierce J., Lalive P.H., Lövblad K.-O., Braillard O. (2022). Long COVID neuropsychological deficits after severe, moderate, or mild infection. Clin. Transl. Neurosci..

[118] Blackmon K., Day G.S., Powers H.R., Bosch W., Prabhakaran D., Woolston D., Pedraza O. (2022). Neurocognitive screening in patients following SARS-CoV-2 infection: Tools for triage. BMC Neurol..

[119] Lynch S., Ferrando S.J., Dornbush R., Shahar S., Smiley A., Klepacz L. (2022). Screening for brain fog: Is the montreal cognitive assessment an effective screening tool for neurocognitive complaints post-COVID-19?. Gen. Hosp. Psychiatry.

[120] Klein J., Wood J., Jaycox J.R., Dhodapkar R.M., Lu P., Gehlhausen J.R., Tabachnikova A., Greene K., Tabacof L., Malik A.A. (2023). Distinguishing features of long COVID identified through immune profiling. Nature.

[121] Altmann D.M., Whettlock E.M., Liu S., Arachchillage D.J., Boyton R.J. (2023). The immunology of long COVID. Nat. Rev. Immunol..

[122] Ruffieux H., Hanson A.L., Lodge S., Lawler N.G., Whiley L., Gray N., Nolan T.H., Bergamaschi L., Mescia F., Turner L. (2023). A patient-centric modeling framework captures recovery from SARS-CoV-2 infection. Nat. Immunol..

[123] Mina Y., Enose-Akahata Y., Hammoud D.A., Videckis A.J., Narpala S.R., O’Connell S.E., Carroll R., Lin B.C., McMahan C.C., Nair G. (2023). Deep phenotyping of neurologic postacute sequelae of SARS-CoV-2 infection. Neurol. Neuroimmunol. Neuroinflamm..

[124] Douaud G., Lee S., Alfaro-Almagro F., Arthofer C., Wang C., McCarthy P., Lange F., Andersson J.L.R., Griffanti L., Duff E. (2022). SARS-CoV-2 is associated with changes in brain structure in UK Biobank. Nature.

[125] Hosp J.A., Reisert M., Dressing A., Gotz V., Kellner E., Mast H., Arndt S., Waller C.F., Wagner D., Rieg S. (2024). Cerebral microstructural alterations in Post-COVID-condition are related to cognitive impairment, olfactory dysfunction and fatigue. Nat. Commun..

[126] Wu L., Zhang Z., Liang X., Wang Y., Cao Y., Li M., Zhou F. (2024). Glymphatic system dysfunction in recovered patients with mild COVID-19: A DTI-ALPS study. iScience.

[127] Greene C., Connolly R., Brennan D., Laffan A., O’Keeffe E., Zaporojan L., O’Callaghan J., Thomson B., Connolly E., Argue R. (2024). Blood-brain barrier disruption and sustained systemic inflammation in individuals with long COVID-associated cognitive impairment. Nat. Neurosci..

[128] Chaganti J., Poudel G., Cysique L.A., Dore G.J., Kelleher A., Matthews G., Darley D., Byrne A., Jakabek D., Zhang X. (2024). Blood brain barrier disruption and glutamatergic excitotoxicity in post-acute sequelae of SARS COV-2 infection cognitive impairment: Potential biomarkers and a window into pathogenesis. Front. Neurol..

[129] VanElzakker M.B., Bues H.F., Brusaferri L., Kim M., Saadi D., Ratai E.M., Dougherty D.D., Loggia M.L. (2024). Neuroinflammation in post-acute sequelae of COVID-19 (PASC) as assessed by [(11)C]PBR28 PET correlates with vascular disease measures. Brain Behav. Immun..

[130] Peluso M.J., Ryder D., Flavell R.R., Wang Y., Levi J., LaFranchi B.H., Deveau T.M., Buck A.M., Munter S.E., Asare K.A. (2024). Tissue-based T cell activation and viral RNA persist for up to 2 years after SARS-CoV-2 infection. Sci. Transl. Med..

[131] Perez D.L., Nicholson T.R., Asadi-Pooya A.A., Begue I., Butler M., Carson A.J., David A.S., Deeley Q., Diez I., Edwards M.J. (2021). Neuroimaging in functional neurological disorder: State of the field and research agenda. Neuroimage Clin..

[132] Mavroudis I., Kazis D., Kamal F.Z., Gurzu I.L., Ciobica A., Padurariu M., Novac B., Iordache A. (2024). Understanding Functional Neurological Disorder: Recent insights and diagnostic challenges. Int. J. Mol. Sci..

[133] Schneider A., Weber S., Wyss A., Loukas S., Aybek S. (2024). BOLD signal variability as potential new biomarker of functional neurological disorders. Neuroimage Clin..

[134] Maurer C.W., LaFaver K., Limachia G.S., Capitan G., Ameli R., Sinclair S., Epstein S.A., Hallett M., Horovitz S.G. (2018). Gray matter differences in patients with functional movement disorders. Neurology.

[135] Begue I., Adams C., Stone J., Perez D.L. (2019). Structural alterations in functional neurological disorder and related conditions: A software and hardware problem?. Neuroimage Clin..

[136] van Campen C., Rowe P.C., Verheugt F.W.A., Visser F.C. (2020). Cognitive function declines following orthostatic stress in adults with myalgic encephalomyelitis/chronic fatigue syndrome (ME/CFS). Front. Neurosci..

[137] van Campen C., Rowe P.C., Verheugt F.W.A., Visser F.C. (2020). Numeric rating scales show prolonged post-exertional symptoms after orthostatic testing of adults with myalgic encephalomyelitis/chronic fatigue syndrome. Front. Med..

[138] van Campen C., Rowe P.C., Visser F.C. (2020). Cerebral blood flow is reduced in severe myalgic encephalomyelitis/chronic fatigue syndrome patients during mild orthostatic stress testing: An exploratory study at 20 degrees of head-up tilt testing. Healthcare.

[139] van Campen C., Rowe P.C., Visser F.C. (2021). Cerebral blood flow remains reduced after tilt testing in myalgic encephalomyelitis/chronic fatigue syndrome patients. Clin. Neurophysiol. Pract..

[140] van Campen C.M.C., Rowe P.C., Visser F.C. (2023). Worsening symptoms is associated with larger cerebral blood flow abnormalities during tilt-testing in myalgic encephalomyelitis/chronic fatigue syndrome (ME/CFS). Medicina.

[141] Blitshteyn S., Verduzco-Gutierrez M. (2024). Long COVID: A major public health issue. Am. J. Phys. Med. Rehabil..

